# Supplementary material for: Carbonized Dehydroascorbic Acid: Aim for Targeted Repair of Graphene Defects and Bridge Connection of Graphene Sheets with Small Size
Source: Nanomaterials (Basel). 2020 Mar 16;10(3):531. doi: 10.3390/nano10030531 (PMC7153701; doi:10.3390/nano10030531)
Supplement: Supplementary file 1 [file nanomaterials-10-00531-s001.pdf]

nanomaterials

Supplementary Materials: Carbonized Dehydroascorbic Acid: Aim for Targeted Repair of Graphene Defects and Bridge Connection of Graphene Sheets with Small Size

Jing Li [1,2,*], Jinfeng Lai [1], Jialiang Liu [1], Rubai Lei [1] and Yuxun Chen [3,*]

1 School of Chemistry and Chemical Engineering, South China University of Technology, Guangzhou 510641, China; laijinfeng666@126.com (Jinfeng Lai); jia.liang.L@outlook.com (Jialiang Liu); leirubai@foxmail.com (R.L.)

2 South China University of Technology-Zhuhai Institute of Modern Industrial Innovation, Zhuhai 519000, China

3 School of Mechanical and Automotive Engineering, South China University of Technology, Guangzhou 510641, China

* Correspondence: ljing@scut.edu.cn (J.L.); yuxunch@scut.edu.cn (Y.C.)

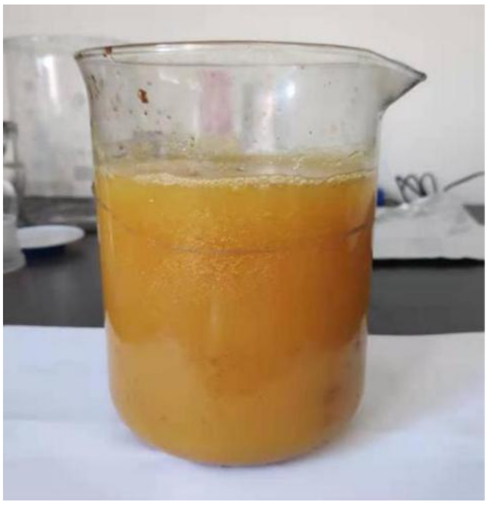

Figure S1. The dispersion solution of GO, obtained after secondary oxidation reaction.

Vitamin C was added to reduce GO:

The reduction of GO by Vitamin C is possibly related to SN2 nucleophilic substitution between hydroxyl and epoxy groups. Vitamin C molecular has enol structure (Figure S2a) and is able to release two protons in aqueous solution to form oxygen negative Vitamin C ions (Figure S2c) so that they may undergo substitution reaction with oxygen-containing groups on GO films (adjacent epoxy and hydroxyl groups) and further heat release reaction to form DHAA (Figure S2b). The route for chemical reduction of GO is shown in Figure S2d,e. As it is shown in Figure S2d, Vitamin C, after dissolved in aqueous solution, release two H^+ to form negatively charged nucleophilic molecules, and these nucleophilic molecules attack the carbenium ions linked together with hydroxyl from its rear end, while a hydroxyl group is integrated with free H^+ to form intermediate α after releasing a H_2O molecule. The second step nucleophilic substitution reaction occurs between oxygen negative Vitamin C ions at its other end and carbenium positive ion of adjacent hydroxyl group, while free hydrogen positive ion is integrated with adjacent hydroxyl to form an intermediate after releasing a H_2O molecule. The intermediate further generates DHAA (Figure S2b) and the initially reduced GO through a heat release reaction at 80 °C. and effectively restores the partially damaged sp_2 structure of graphene. The reduction process of epoxy group is similar to that of hydroxyl group, and the reaction pathway is shown in Figure S2e. The nucleophilic molecules attack the carbocation attached to the epoxy group at its rear end by a two-step SN2 nucleophilic reaction to detach a small molecule H_2O to form the intermediate. Then the initial reduced GO is obtained by further thermal reduction elimination reaction.

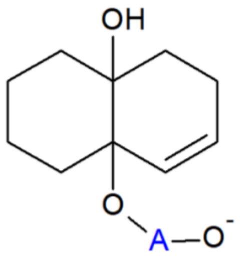

Intermediate *α*

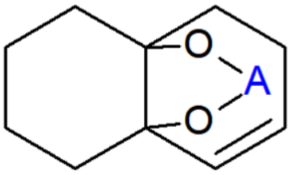

Intermediate *β*

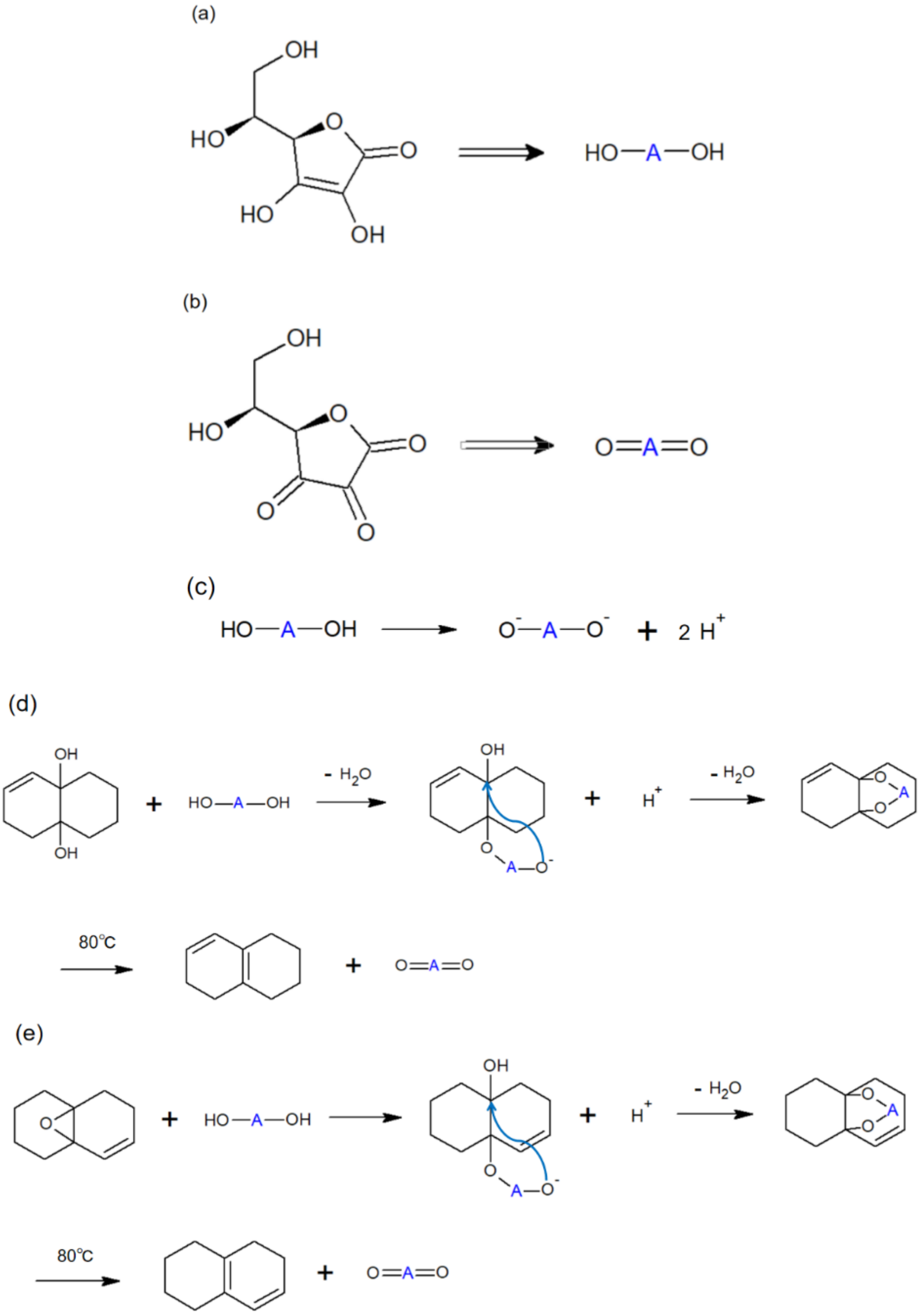

Figure S2. The route of reaction for the partial reduction of GO by oxygen negative Vitamin C ions.

nanomaterials

MDPI

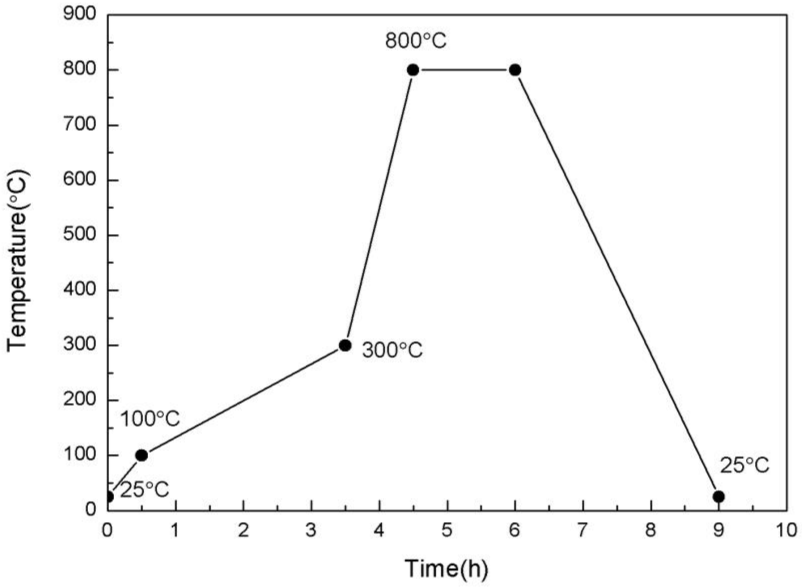

Figure S3. Procedure temperature reduction rGO/VC films.

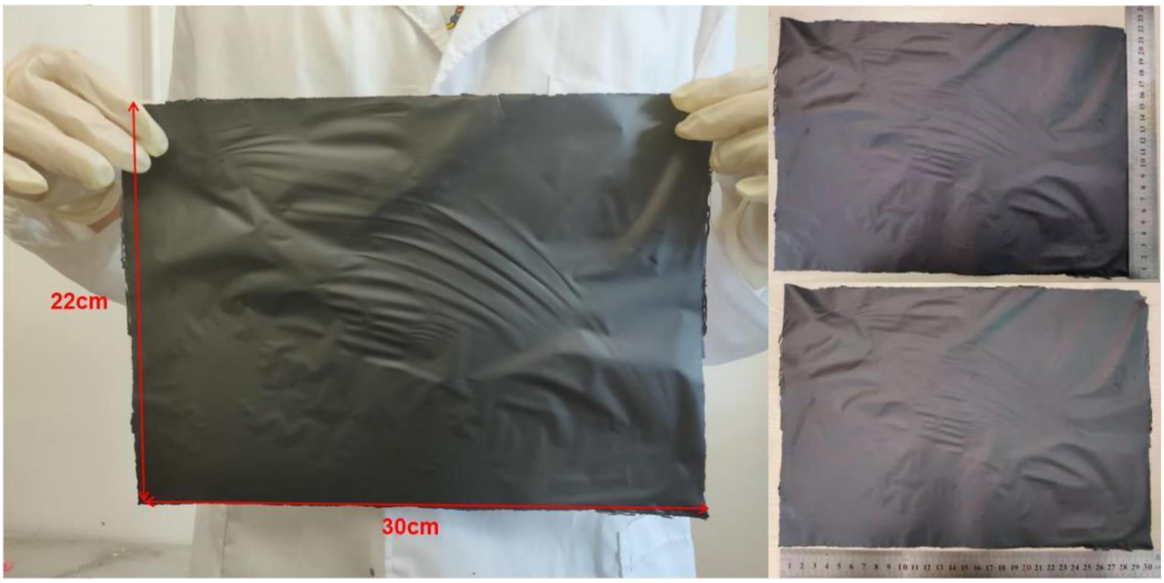

Figure S4. GO/VC films with a size of 22 × 30 cm.

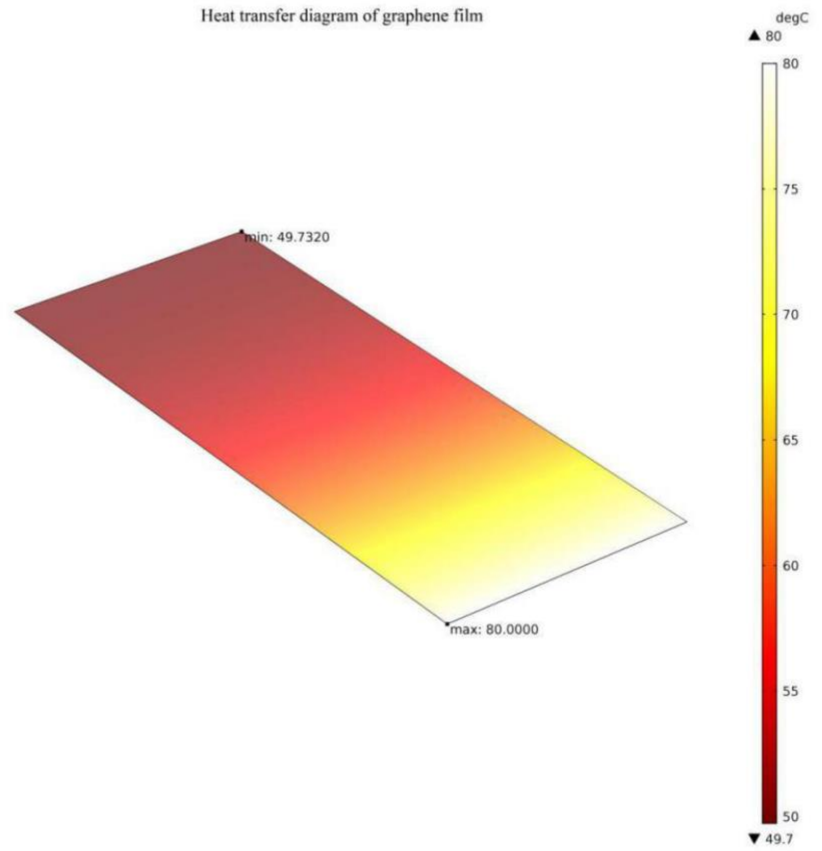

Figure S5. Heat transfer simulation results of rGO/VC-15% films with COMSOL multiphysical (Multiphysics 5.3a, COMSOL, Stockholm, Sweden) field simulation software.

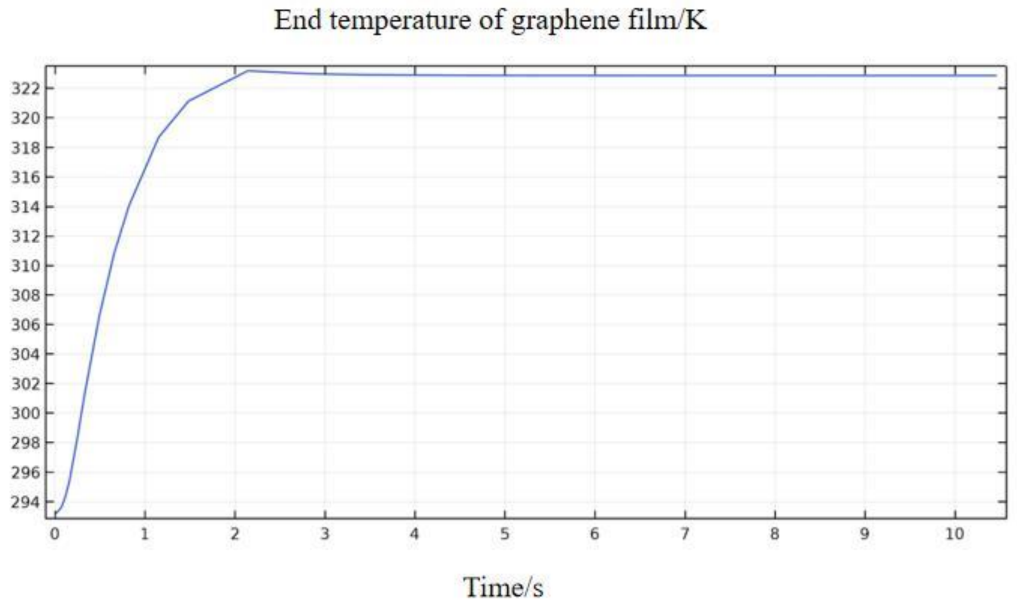

Figure S6. The change of terminal temperature of rGO/VC-15% film during heat transfer simulation test.

Table S1. The thickness and the bulk density of samples in the LFA 447 Nanoflash measurement.

Sample	Thickness (μm)	Bulk density (g cm^{-3})	Thermal diffusivity(mm^2/s)	Specific Heat Capacity (J g^{-1} K^{-1})
rGO	14	0.72	1062.944	0.70
rGO/VC-5%	15	0.76	1514.526	0.70
rGO/VC-10%	14	0.83	1550.381	0.70
rGO/VC-15%	15	0.89	1654.831	0.70
rGO/VC-20%	16	0.87	1523.648	0.70
rGO/VC-25%	16	0.9	1417.523	0.70
